# Investigation of Daily Glucose Profile of Inpatients in Non-endocrinology Departments in Chinese Population

**DOI:** 10.3389/fpubh.2020.521227

**Published:** 2020-11-05

**Authors:** Xiaoyang Sun, Minghui Gui, Huiqun Huang, Huihua Zhao, Hongmei Yan, Hua Bian, Xin Gao

**Affiliations:** ^1^Department of Endocrinology, Zhongshan Hospital, Fudan University, Shanghai, China; ^2^Institute of Metabolic Disease, Fudan University, Shanghai, China

**Keywords:** diabetes, blood glucose, point-of-care systems, hyperglycemia, hypoglycemia

## Abstract

**Background:** Inpatient hyperglycemia is associated with poor prognosis and increased hospitalization expenses. China has a large population of inpatients with hyperglycemia, but their glucose monitoring states (including preprandial, postprandial and bedtime glucose) are unknown, especially in non-endocrinology departments.

**Methods:** In this cross-sectional study, 5,790 patients with hyperglycemia from 31 non-endocrinology departments were enrolled, and a total of 1,22,032 point-of-care blood glucose (POC-BG) records were collected. The “patient-day” unit of measure was used as a metric for the inpatient glucose. A total of 2,763 patients from endocrinology wards were included for the comparison of the improvement of glycemic management during hospitalization in non-endocrinology wards.

**Results:** A total of 61.16% of patient-days had <4 POC-BG tests. Postprandial POC-BG was tested significantly less frequently than preprandial POC-BG (10.60% vs. 58.85% of all records, *P* < 0.001). The patient-day-weighted mean BG was higher in non-ICU wards than in the ICU (9.72 ± 3.37 vs. 9.00 ± 3.19 mmol/L, *P* < 0.001). The rate of hyperglycemia (BG >10 mmol/L) was 37.60% in all non-endocrinology wards (ICU vs. non-ICU: 33.19% vs. 39.17%, *P* < 0.001). In non-ICU wards, the rate of hyperglycemia (BG >10 mmol/L) was significantly higher in surgical wards than in medical wards (40.30% vs. 36.90%, *P* < 0.001). ICU had a significantly higher rate of achieving the blood glucose target than the non-ICU wards (32.50% vs. 26.38%, *P* < 0.001). In the non-ICU departments, medical wards had higher rate of achieving the blood glucose target than surgical wards (39.70% vs. 19.08%, *P* < 0.001). With increasing days of hospitalization, there was no improvement in glycemic control in non-endocrinology wards. The ICU had a significantly higher rate of hypoglycemia than non-ICU wards (4.62% vs. 3.73%, *P* < 0.05). In non-ICU wards, medical wards had a significantly higher rate of hypoglycemia than surgical wards (5.71% vs. 2.75%, *P* < 0.05).

**Conclusions:** Both the frequency of BG monitoring and the daily glucose profile of inpatients in Chinese non-endocrinology departments were less than ideal and need to be urgently improved.

## Introduction

The prevalence of diabetes is as high as 11.6% in the Chinese population (1). The prevalence of diabetes is even higher (17.8%) among inpatients in general hospitals. However, approximately five-sixths of inpatients with diabetes are admitted to non-endocrinology departments, where glucose management may not be the primary focus of practitioners. Besides known diabetes, hyperglycemia may be caused by newly diagnosed diabetes or illness-related stress. Studies show that inpatient hyperglycemia, regardless of a history of diabetes, is associated with poor prognosis and death (2). Poor glycemic control is closely related to an increase in infection (3), cardiovascular events (4), mortality and hospitalization expenses (5). Thus, improving glycemic management in both diabetic and non-diabetic inpatients is a matter of great urgency. Therefore, investigating the state of inpatient glucose monitoring in non-endocrinology departments is very important to assess and improve glycemic management in China.

Bedside point-of-care blood glucose (POC-BG) measurement is the most commonly used technique for inpatient glucose monitoring in China. Compared to plasma glucose, POC-BG can be easily tested multiple times per day and is better at showing the daily glucose profile of inpatients. More importantly, it is also the main basis on which practitioners make therapeutic decisions about glycemic management (6). However, despite some studies (7, 8) about inpatient glycemic management in non-endocrinology wards, few studies have used POC-BG to show the glucose profile throughout the hospitalization of inpatients in China. Data on overall inpatient glucose monitoring in China are still scarce. Therefore, the objective of this study is to investigate glucose monitoring in the Chinese population in non-endocrinology departments by means of POC-BG measurements. The daily glucose profiles (including the preprandial, postprandial and bedtime BG) of inpatients in various non-endocrinology wards (including surgical wards, medical wards and ICU) will be shown.

## Methods

### Ethics Statement

The study design was in accordance with the Helsinki Declaration of 1975 and approved by the ethics committee of the Fudan University Zhongshan Hospital, and each subject provided written informed consent.

### Participants

The investigation was carried out at Zhongshan Hospital, Fudan University, which is one of the largest general hospitals in Shanghai China. From October 2015 to April 2017, a total of 5,790 inpatients were enrolled to investigate glucose monitoring in non-endocrinology departments. Another group of 2,763 patients from the endocrinology ward were included for comparison of the improvement of glycemic management during hospitalization in non-endocrinology wards. The inclusion criteria were as follows: patients who both (1) with hyperglycemia (plasma glucose levels higher than 7.8 mmol/L at any time during hospitalization or patients who had a history of type 2 diabetes) and (2) had point-of-care BG tests during hospitalization. The exclusion criteria were as follows: (1) <18 years of age; (2) patients who had a diagnosis of pregnancy or mental disorders. According to the departments to which they were admitted, participants in non-endocrinology wards were divided into groups of surgical wards (*n* = 3,076), medical wards (*n* = 1,331), and ICUs (*n* = 1,383).

### Data Measurement and Collection

The POC-BG records from all the wards surveyed were collected and transmitted by a unified information system (GLUPAD®, Sinomedisite Inc., Beijing, China). The system includes a glucose measuring device and POC-BG data analysis software that was automatically connected to the hospital information system (HIS). Preprandial BG levels were measured half an hour before regular meals, while postprandial BG levels were measured 2 h after 3 daily meals. Bedtime BG levels were tested before the patient slept. As this was a real-world study, the personalized frequency of POC-BG tests was decided based on the reasons for admission and the participants' glucose metabolic condition, as well as clinical feasibility.

Serum parameters such as fasting plasma glucose (FPG), serum alanine aminotransferase (ALT), aspartate aminotransferase (AST), alkaline phosphatase (ALP), total cholesterol (TC), high-density lipoprotein cholesterol (HDL-C), triglyceride (TG), creatinine (Cr), blood urea nitrogen (BUN) and uric acid (UA) levels were measured by a model 7600 automated bioanalyzer (Hitachi, Tokyo, Japan). Low-density lipoprotein cholesterol (LDL-C) was calculated by the Friedewald equation. Hemoglobin A1c (HbA1c) were measured via high performance liquid chromatography.

### Statistical Analysis

The frequency of BG monitoring, the rate of hyper- and hypoglycemia, and the rate of achieving the blood glucose target were calculated for intensive care units (ICUs) and non-ICU wards (including surgical and medical wards) separately. “Patient-day,” which means per patient per hospitalization day, was applied as the analyzing unit because it could most faithfully reflect the quality of inpatient glycemic control (9). Patient-day averages were calculated as previously reported (10). These patient-day averages were then aggregated to the group level, and then mean BG levels, the hyperglycemia rates, hypoglycemia rates and rates of achieving the blood glucose target were compared among groups. When calculating the rate of hyperglycemia, 10 mmol/L (180 mg/dl) was used as a cut-off point for hyperglycemia, as recommended by the ADA/AACE guidelines (11). In particular, the rate of hyperglycemia over different cut-off points (the percentage of patient-days with mean BG >8, >10, >12, >14, >16, >18, and >20 mmol/L) was assessed. Individual blood glucose targets were determined by participants' history of hyperglycemia and other recorded comorbidities, as well as the risk of hypoglycemia, with a consensus statement on inpatient glycemic control in Chinese adults (12) as a reference. The rate of achieving the blood glucose target was calculated as the percentage of patient-days with both mean preprandial and postprandial POC-BG within the target range. For hypoglycemia, the percentage of patient-days with at least one POC-BG <3.9 mmol/L was calculated. We also assessed the differences between groups in the rates of preprandial hyperglycemia (>8 mmol/L) on different days of hospitalization in surgical wards and medical wards using POC-BG data from the Department of Endocrinology in the study period as a reference. The rates of hyperglycemia (>10 mmol/L) on different days of hospitalization in the ICU were also calculated.

Age and blood glucose, hemoglobin A1c, total cholesterol, triglyceride, high-density lipoprotein cholesterol, low-density lipoprotein cholesterol and uric acid levels were normally distributed and are presented as the mean ± SD. Multigroup comparisons of these data were analyzed using one-way ANOVA after testing for homogeneity of variances, followed by Bonferroni's test for comparisons between every two-group combination. Triglyceride, alanine aminotransferase, aspartate aminotransferase, alkaline phosphatase, creatinine and blood urea nitrogen levels were non-normally distributed data, which are shown as the median (25th to 75th percentile). Multigroup comparisons of these data were analyzed using the Kruskal-Wallis test, and the Mann-Whitney *U*-test with the Bonferroni-corrected *p*-value was used for comparisons between every two-group combination. For categorical data such as the percentage of male participants or the rate of hyperglycemia and hypoglycemia, a chi-square test or a partitioned chi-square test was applied. No further adjustment was made for multivariable comparisons. Therefore, the results should be considered exploratory. To estimate differences between groups for the rates of preprandial hyperglycemia (>8 mmol/L) on different days of hospitalization in surgical wards, medical wards and the endocrinology departments, the generalized estimating equation (GEE) mode was used. The unstructured covariance structure was used to model the correlation of tests from the same patients. The difference in the preprandial hyperglycemia rate between the first and sixth days in 3 groups was compared using the chi-square test. The rate of preprandial hyperglycemia on the sixth day was also compared among the 3 groups using the partitioned chi-square test.

Calculations were made using SPSS version 20.0 (SPSS Inc., Chicago, IL, USA), GraphPad Prism version 7.00 (GraphPad Software, San Diego, CA) and R version 3.6.2. *P*-values of <0.05 were considered statistically significant.

## Results

### Characteristics of the Study Participants

A total of 5,790 patients from 31 non-endocrinology departments (including 12 surgical wards, 12 medical wards, and 7 ICU settings) were surveyed, and a total of 2,763 patients from the endocrinology ward were included for comparison. A total of 2,30,985 POC-BG records were analyzed, of which 59,856 were from surgical wards, 30,054 were from medical wards, 32,122 were from the ICU, and 1,08,953 were from the endocrinology ward. The demographic and biochemical characteristics of the enrolled patients are shown in Table 1.

**Table 1:** Demographic and biochemical characteristics of the hospitalized patients, according to wards.

	**Total (*n* = 8,553)**	**Surgical ward (*n* = 3,076)**	**Medical ward (*n* = 1,331)**	**ICU (*n* = 1,383)**	**Endocrinology ward (*n* = 2,763)**	***p-*value**
Male (%)	5093 (59.55%)	1468 (53.13%)	872 (65.51%)	1916 (62.29%)	837 (60.52%)	<0.001
Age (years)	60.19 ± 14.13	61.7 ± 12.06	61.09 ± 14.77	62.59 ± 15.62^†^	56.89 ± 14.57^*^^†^^Δ^	<0.001
FBG (mmol/L)	7.21 ± 3.16	7.15 ± 3.09	6.89 ± 3.22	6.93 ± 3.01	7.45 ± 3.22^*^	<0.001
HbA1c (%)	7.34 ± 2.09	6.7 ± 1.66	6.63 ± 1.62	6.56 ± 1.67	8.05 ± 2.25^*^	<0.001
TC (mmol/L)	4.22 ± 1.21	4.13 ± 1.15	4.22 ± 1.34	4.11 ± 1.33	4.31 ± 1.17^*^^Δ^	<0.001
TG (mmol/L)	1.26(0.9–1.83)	1.22 (0.88–1.73)	1.28 (0.91–1.8)	1.13 (0.81–1.63)	1.34 (0.93–1.96)^*^	<0.001
HDL-c (mmol/L)	1.08 ± 0.36	1.07 ± 0.35	1.06 ± 0.38	1.06 ± 0.44	1.11 ± 0.34^*^	<0.001
LDL-c (mmol/L)	2.44 ± 0.98	2.41 ± 1.01	2.48 ± 1.13	2.39 ± 0.94	2.45 ± 0.9	0.263
ALT (U/L)	20 (12–35)	23 (13–44)	19 (12–32)^*^	24 (13–44.5)^*^^†^	18 (12–29)^*^^Δ^	<0.001
AST (U/L)	20 (15–29)	23 (17–34)	20 (15–29)	23 (17–35)^*^^†^	18 (14–24)^*^^Δ^	<0.001
ALP (U/L)	71 (57–92)	75 (59–101)	72 (58–97)	77 (57–111)	67 (55–81)^*^	<0.001
Cr (umol/L)	70 (57–87)	70 (58–88)	73 (61–98)^*^	72 (55–95)^*^^†^	68 (56–81)^*^^†^^Δ^	<0.001
BUN (mmol/L)	5.4 (4.3–7.2)	5.6 (4.2–7.7)C	5.5 (4.3–7.9)^*^	5.9 (4.1–9.78)^*^^†^	5.3 (4.3–6.5)^*^^†^^Δ^	<0.001
UA (μmol/L)	293.5 ± 123.74	262.06 ± 128.47	313.51 ± 127.45^*^	289.19 ± 163.79^*^^†^	315.42 ± 99.81^*^^Δ^	<0.001

*ICU, intensive care unit; FBG, fasting blood glucose; HbA1c, hemoglobin A1c; TC, total cholesterol; TG, triglyceride; HDL-c, high density lipoprotein cholestrol; LDL-c, low density lipoprotein cholestrol; ALT, alanine aminotransferase; AST, aspartate aminotransferase; ALP, alkaline phosphatase; Cr, creatinine; BUN, blood urea nitrogen; UA, uric acid*.

*Data are presented as the means±SD, percentages or medians (25th to 75th percentiles)*.

* *p < 0.05/6 for vs. surgical wards*.

† *p < 0.05/6 for vs. medical wards*.

Δ *p < 0.05/6 for vs. ICU*.

### Frequency of BG Monitoring in Non-endocrinology Wards

**Figure 1:**
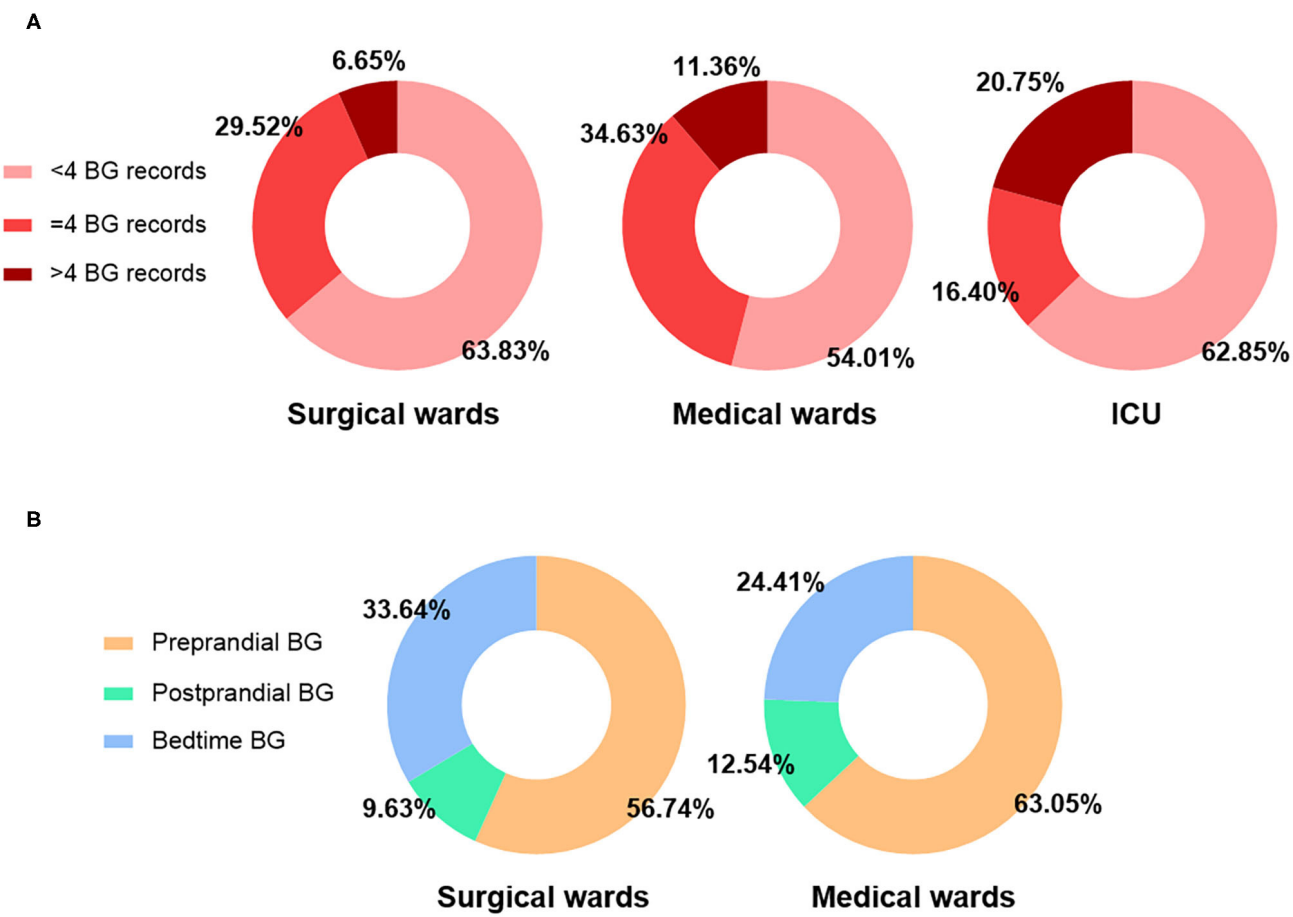
Frequency of BG monitoring. **(A)** Proportions of patient-days with POC-BG testing <4, =4 and >4 times in surgical wards, medical wards, non-ICU wards (surgical + medical wards) and the ICU. **(B)** Proportions of preprandial, postprandial and bedtime POC-BG of all records in surgical wards, medical wards and non-ICU wards (surgical + medical wards).

The frequency of BG monitoring is shown in Figure 1A. In all non-endocrinology wards, an average of 3 (2-4) POC-BG tests were performed per patient-day. A total of 61.16% of patient-days had POC-BG testing <4 times, 27.34% of patient-days had POC-BG testing 4 times, and only 11.50% had POC-BG testing >4 times. The ICU had a significantly higher percentage of patient-days with >4 POC-BG tests than the non-ICU wards (20.75% vs. 8.22%, *P* < 0.001). The percentages of POC-BG records tested at different times are shown in Figure 1B. The percentages of patients with at least one preprandial, postprandial and bedtime POC-BG record were 72.95, 30.20, and 73.59%, respectively, in the non-ICU wards. These results all suggested that the frequency of POC-BG monitoring, especially postprandial BG monitoring, was relatively low in non-endocrinology wards.

### Rate of Hyperglycemia in Non-endocrinology Wards

The patient-day-weighted mean BG was higher in the non-ICU (mmol/L) than in the ICU (9.72 ± 3.37 vs. 9.00 ± 3.19 mmol/L, *P* < 0.001) wards. In the non-ICU settings, the mean BG in surgical wards was significantly higher than that in medical wards (9.78 ± 3.27 mmol/L vs. 9.61 ± 3.57 mmol/L, *P* < 0.001).

The percentage of patient-days with mean BG >10 mmol/L was 37.60% in all non-endocrinology wards; ICUs had a significantly lower percentage than non-ICU wards (33.19% vs. 39.17%, *P* < 0.001). In the non-ICU departments, the percentage was significantly higher in surgical wards than in medical wards (40.30% vs. 36.90%, *P* < 0.001). In addition, 56.85% of patient-days in all non-endocrinology wards had at least one recorded BG > 10 mmol/L. The ICU also had a significantly lower percentage of hyperglycemia than the non-ICU wards (48.44% vs. 59.84%, *P* < 0.001). In the non-ICU, there was no significant difference in hyperglycemia percentages between surgical wards and medical wards (60.29% vs. 58.94%, *P* > 0.05).

**Figure 2:**
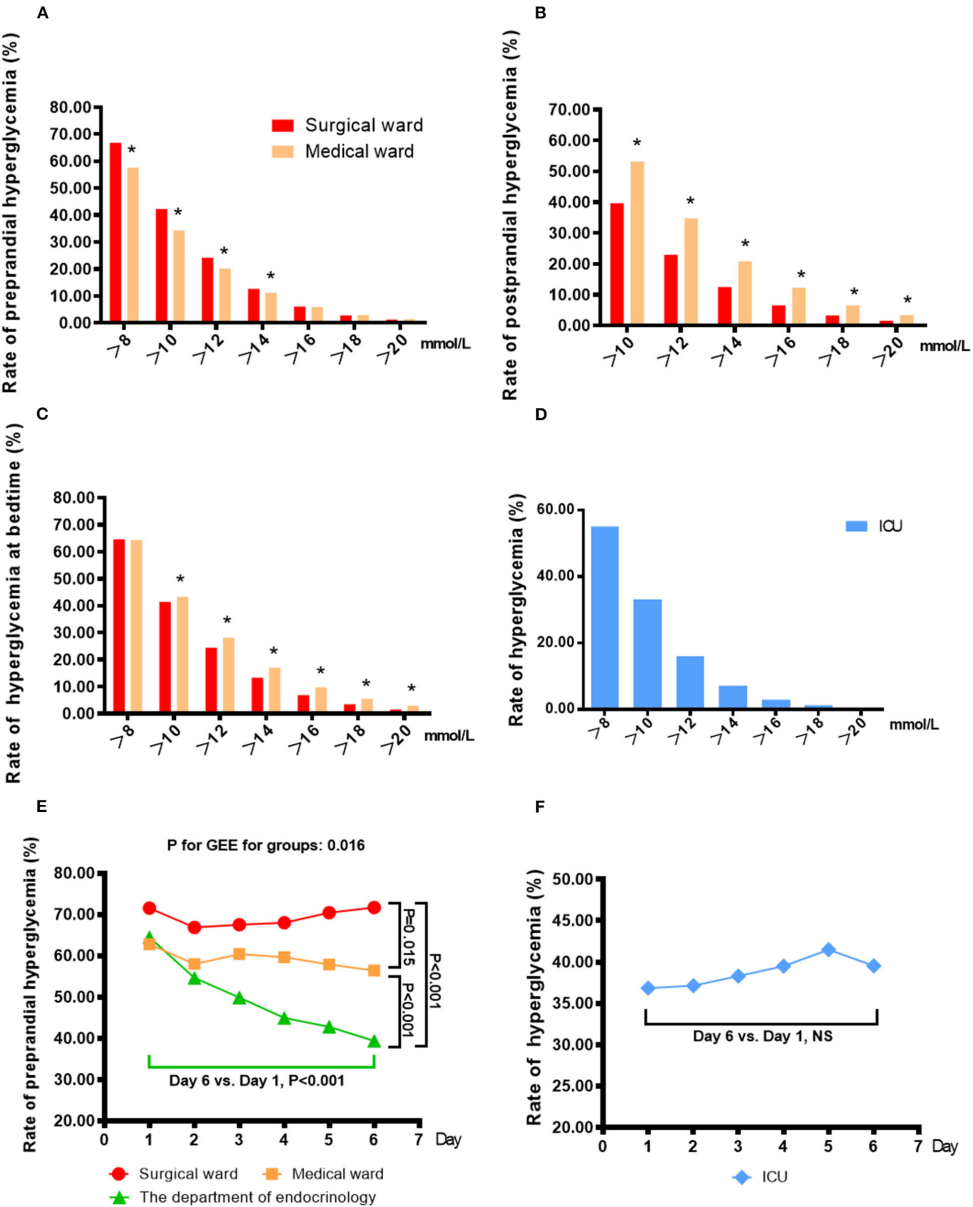
Percentage of patient-days in which patient-day-weighted mean BG exceeded different cut-off points in the non-ICU wards **(A–C)** and the ICU **(D)** and the changes in the rates of hyperglycemia with increasing days of hospitalization in the non-ICU wards **(E)** and the ICU **(F)**. **(A)** The rates of preprandial hyperglycemia were 66.87, 42.29, 24.19, 12.65, 6.11, 2.77, and 1.25% in surgical wards, respectively, for each cut-off point (>8, >10, >12, >14, >16, >18, and >20 mmol/L), and the rates were 57.58, 34.35, 20.14, 11.18, 5.91, 2.98, and 1.43% in medical wards. *p < 0.05 vs. surgical wards. **(B)** The rates of postprandial hyperglycemia were 39.69, 22.90, 12.47, 6.58, 3.28, and 1.56% in surgical wards, respectively, for each cut-off point (>10, >12, >14, >16, >18, and >20 mmol/L), and the rates were 53.18, 34.78, 20.80, 12.24, 6.52, and 3.38% in medical wards. *p < 0.05 vs. surgical wards. **(C)** The rates of hyperglycemia at bedtime were 64.63, 41.46, 24.46, 13.26, 6.79, 3.44, and 1.54% in surgical wards, respectively, for each cut-off point (>8, >10, >12, >14, >16, >18, and >20 mmol/L), and the rates were 64.35, 43.28, 28.06, 17.00, 9.73, 5.47, and 2.96% in medical wards. *p < 0.05 vs. surgical wards. **(D)** Proportions of patient-days with the average whole-day BG above different cut-off points in the ICU. The rates of hyperglycemia were 55.11, 33.19, 16.01, 7.15, 2.99, 1.29, and 0.64%, respectively, for each cut-off point (>8, >10, >12, >14, >16, >18, and >20 mmol/L). **(E)** The rates of preprandial hyperglycemia (>8 mmol/L) on different days of hospitalization in surgical wards, medical wards and the endocrinology departments. *(F) The rates of hyperglycemia (>10 mmol/L) on different days of hospitalization in the ICU.

Moreover, for non-ICU wards, the rates of hyperglycemia at different times using different cut-off points are shown in Figures 2A–C. The rates of preprandial hyperglycemia were 66.87, 42.29, 24.19, 12.65, 6.11, 2.77, and 1.25% in surgical wards, respectively, for each cut-off point (>8, >10, >12, >14, >16, >18, and >20 mmol/L). The rates were 57.58, 34.35, 20.14, 11.18, 5.91, 2.98, and 1.43% in medical wards. Surgical wards had a significantly higher rate of preprandial hyperglycemia than medical wards (>8, >10, >12, and >14 mmol/L, *P* < 0.05 for all). The rates of postprandial hyperglycemia were 39.69, 22.90, 12.47, 6.58, 3.28, and 1.56% in surgical wards, respectively, for each cut-off point (>10, >12, >14, >16, >18, and >20 mmol/L). The rates were 53.18, 34.78, 20.80, 12.24, 6.52, and 3.38% in medical wards, and surgical wards had a significantly lower rate of postprandial hyperglycemia than medical wards (>8, >10, >12, >14, >16, >18, and >20 mmol/L, *P* < 0.05 for all). The rates of hyperglycemia at bedtime were 64.63, 41.46, 24.46, 13.26, 6.79, 3.44, and 1.54% in surgical wards, respectively, for each cut-off point (>8, >10, >12, >14, >16, >18, and >20 mmol/L). The rates were 64.35, 43.28, 28.06, 17.00, 9.73, 5.47, and 2.96% in medical wards, and surgical wards had a significantly lower rate of hyperglycemia at bedtime than medical wards (>10, >12, >14, >16, >18, and >20 mmol/L, *P* < 0.05 for all). Proportions of patient-days with the whole-day average BG above different cut-off points in the ICU are shown in Figure 2D. The rates of hyperglycemia in the ICU were 55.11, 33.19, 16.01, 7.15, 2.99, 1.29, and 0.64%, respectively, for each cut-off point (>8, >10, >12, >14, >16, >18, and >20 mmol/L). In accordance with the lower rate of hyperglycemia, the ICU had a significantly higher rate of achieving the blood glucose target than the non-ICU wards (32.50% vs. 26.38%, *P* < 0.001). In the non-ICU departments, medical wards had higher rate of achieving the blood glucose target than surgical wards (39.70% vs. 19.08%, *P* < 0.001).

To observe the improvement of glycemic management during hospitalization in various wards, we analyzed the changes in the rate of hyperglycemia with increasing days of hospitalization (Figures 2E,F). In the Department of Endocrinology, the rate of preprandial hyperglycemia (>8 mmol/L) on the sixth hospitalization day was significantly lower than that on the first day (39.40% vs. 64.50%, *P* < 0.05), but there was no significant difference in the rate in other non-ICU wards (71.55% vs. 71.70% in surgical wards, 62.81% vs. 56.43% in medical wards). On the first day of hospitalization, there were no significant differences in the rates of preprandial hyperglycemia in all non-ICU wards (*P* > 0.05). On the sixth day of hospitalization, the rate of hyperglycemia in the Department of Endocrinology was significantly lower than that in surgical and medical wards (39.40% vs. 71.70% for surgical wards, *P* < 0.001; vs. 56.43% for medical wards, *P* < 0.001). Although the ICU had a generally low rate of hyperglycemia (>10 mmol/L), no significant improvement was found with increasing days of hospitalization (39.55% vs. 36.85%, *P* > 0.05).

### Rate of Hypoglycemia

The rate of hypoglycemia was 3.97% in all non-endocrinology departments surveyed. The ICU had a significantly higher rate of hypoglycemia than the non-ICU wards (4.62% vs. 3.73%, *P* < 0.05). In the non-ICU departments, medical wards had a significantly higher rate of hypoglycemia than surgical wards (5.71% vs. 2.75%, *P* < 0.05). Hypoglycemic events were mainly reported before meals (60.01%), but 27.12% of hypoglycemic events were observed at bedtime, and 12.87% were observed after meals.

## Discussion

The state of overall inpatient glucose monitoring in China has not yet been determined. From the study, we found that both the frequency of BG monitoring and the daily glucose profile of inpatients in non-endocrinology departments in China were less than ideal. In all non-endocrinology wards, each patient-day had an average of 3 (2-4) POC-BG tests. Postprandial records only account for 10.60% of all POC-BG records. The rates of preprandial hyperglycemia (>8 mmol/L) and postprandial hyperglycemia (>10 mmol/L) in non-ICU wards were 63.65% and 44.18%, respectively. The rate of hyperglycemia (>12 mmol/L) was 16.01% in the ICU. The ICU had a significantly higher rate of achieving the personalized blood glucose target than the non-ICU wards (32.50% vs. 26.38%, *P* < 0.001). Compared with that in the Department of Endocrinology, no significant improvement of glycemic control could be seen in other wards as hospitalization days increased. The rate of hypoglycemia was relatively low, 1.63% in non-ICU wards and 2.02% in the ICU.

In most cases, inpatients in non-endocrinology wards received no more than 4 POC-BG tests during one hospitalization day. In addition, the frequency of postprandial BG monitoring was much lower than the testing frequency at other times in non-ICU wards. This finding may reveal practitioners' insufficient awareness of the importance of postprandial BG monitoring. A previous study (13) reported that the 2 h postprandial plasma glucose test was also less frequently used in non-endocrinology departments in China. However, postprandial glucose plays an important role in glycemic control. Patients achieving the target postprandial BG were more likely to achieve the HbA1c target than those who only met the FPG target (14). Therefore, the monitoring of POC-BG, especially postprandial BG, urgently needs to be improved in non-endocrinology departments.

In the ICU, the mean BG level and the rate of hyperglycemia were still lower than those in the non-ICU wards, although different guidelines (11, 15, 16) all recommend less stringent standards for glycemic control in the ICU. This outcome was similar to those of previous studies (17, 18). However, the ICU had a lower rate of hyperglycemia than the non-ICU wards. This is probably because the practitioners in the ICU attached more importance to avoiding hyperglycemia, and the ICU patients were mainly treated with intravenous insulin, which was easy to adjust for better BG control. However, the detailed insulin information was not available in our study.

When >10 mmol/L was used as the cut-off point for determining hyperglycemia, the rates of hyperglycemia varied dramatically from one study to another. In the United States, the results of a large survey showed that the percentage of patient-days with mean BG >10 mmol/L was 32.2% in the ICU and 32.0% in the non-ICU wards (19). In Brazil, a nationwide survey showed that the percentage of patient-days with at least one BG >10 mmol/L was 61.3% in the ICU and 64.7% in the non-ICU wards (17), and this measure was up to 81.3% in Singapore (20). In our results, the percentage of patient-days with a mean BG >10 mmol/L was 33.19% in the ICU and 39.17% in the non-ICU wards. The percentage of patient-days with at least one BG >10 mmol/L was 48.44% in the ICU and 59.84% in the non-ICU wards. The differences between our outcomes and others could be mainly due to the differences in the quality of local medical care. Other possible factors may be the differences in computational methods, the differences in sample sizes and the differences in geographic and ethnic factors. Moreover, patients admitted into different wards would mean different targets, while little research has been done in the rates of achieving the personalized blood glucose targets in non-endocrinology wards. The current study found that the rate of achieving the personalized blood glucose targets were both less optimistic in ICU (32.50%) and non-ICU (26.38%) wards, which deserved more attention.

Compared with that in the Department of Endocrinology, no significant improvement of glycemic control could be seen in other wards as hospitalization days increased. This may be due to the insufficient experience and awareness of glycemic management. Studies (21, 22) in the United States showed that a lack of knowledge (especially regarding insulin therapy) was the most commonly cited barrier to balanced inpatient glycemic control, which was in accordance with our pilot study of small sample in non-endocrinology doctors and nurses. In our study, the poor outcome of inpatient glucose management may also be at least in part attributed to the lack of skills.

Therefore, protocols for the treatment of hyperglycemia should be formulated and used in non-endocrinology departments.

Compared with hyperglycemia, hypoglycemia was relatively infrequent during the hospitalization period. In our study, the rate of hypoglycemia was 3.97%, which was lower than (17–19, 23) or similar (21, 24) to that in previous studies worldwide. Intensive insulin therapy may be the major cause of hypoglycemia (25). Considering the high frequency of hyperglycemia, the low incidence of hypoglycemia may not be an optimistic outcome but a consequence of loose glycemic control. Hypoglycemia is strongly associated with an increased risk of cardiovascular disease in patients with diabetes (26). Many studies have shown that hypoglycemia is also associated with in-hospital mortality (27). Concerns about these factors may be the cause of inadequate glucose intervention (28). Even so, 27.12% of hypoglycemic events were observed at bedtime. When the bedtime BG was below 6 mmol/L, the risk of nocturnal hypoglycemia was 80% (29). It was reported that 14.9% of diabetic patients experienced nocturnal hypoglycemia during hospitalization in China (25). Therefore, the monitoring of bedtime BG needs to be enhanced to prevent hypoglycemia.

To the best of our knowledge, this study is the first to use POC-BG readings to show the glucose monitoring (including the frequency and the outcomes) of inpatients in a variety of non-endocrinology departments in China. It is also the first to show the daily glucose profile (including the preprandial, postprandial and bedtime BG) of non-endocrinology inpatients in the world. Despite all these findings, the following limitation of our study should be considered: the information of anti-diabetic medication was not well recorded in most non-endocrinology wards in study period. In this way we could not collect anti-diabetic protocols for a large number of patients to investigate the effect of different anti-diabetic medication on inpatient glucose control, which requires future studies.

## Conclusion

POC-BG data collected and transmitted by a unified information system could help to monitor the status of inpatient glycemic management in non-endocrinology departments. As the data showed, the frequency of POC-BG monitoring, especially postprandial BG monitoring, was relatively low in non-endocrinology wards. The glycemic control of non-endocrinology inpatients is less than ideal in China and urgently needs to be improved.

## Data Availability Statement

All datasets generated for this study are included in the article/supplementary material.

## Ethics Statement

The studies involving human participants were reviewed and approved by the ethics committee of the Fudan University Zhongshan Hospital. The patients/participants provided their written informed consent to participate in this study.

## Author Contributions

XG, HB, and HY: research conduction and study supervision. XS and MG: statistical analyses and interpretation of the data. XS: wrote the paper. HH and HZ: performed examination. All authors contributed to the article and approved the submitted version.

## Conflict of Interest

The authors declare that the research was conducted in the absence of any commercial or financial relationships that could be construed as a potential conflict of interest.
